# Tyler Medical Society

**Published:** 1896-09

**Authors:** E. A. Woldert

**Affiliations:** President Tyler Medical Society; Tyler, Texas


### Tyler Medical Society.

---

The JOURNAL is in receipt of the following note from Dr. E. A. Woldert, President of the Tyler Medical Society:

TYLER, TEXAS, August 15, 1896.

*Editors Texas Medical Journal:*

I take pleasure in informing you that the following resolutions, introduced by me, were adopted:

WHEREAS, Every State in this Union, except New Hampshire, have medical laws; and

WHEREAS, Certain contingencies have arisen which have a tendency to defeat proper medical legislation in this State; therefore, be it

*Resolved, 1st*, By the Tyler Medical Society, that we take the initiative in this matter, and that we hereby endorse the medical bill adopted at the last meeting of the Texas State Medical Association held in Forth Worth in April, 1896.

*Resolved, 2nd*, That this society procure copies of this bill and place in the hands of our representatives for study, and urge their persistent support of this measure in order that the constitutional rights of man, "life, liberty, health and the pursuit of happiness may be protected,"

A word in explanation: In giving endorsement to this bill understand that the prime object in doing so is to secure a medical bill to protect the lives of humanity.

Personally I do not entirely approve of the one board idea, because all schools would be required to be represented at the same meeting and at the same time that each examination is to be held, where each would necessarily have to come in mutual contact with the other.

After giving a license to the applicant, regulars would become more responsible for the subsequent action of those who obtain the license from the one board, because according to the provision of the bill there are more regulars on the board than either the homeopaths or eclectics.

In my judgment the three distinct boards idea, subject to a still higher tribunal, would be best, for in that case the meetings for granting licenses would not necessarily be at the same time or place, and the higher tribunal would grant the license and be responsible for such action.

Again, I am not in favor of this provision in the bill: Article 3rd. "The said board shall consist of men *learned* in medicine and surgery." Men who are not graduates should not license graduates.

At the same time, while I hold to such convictions, I shall surrender them for the time being in order to secure a bill, for the above reason.

Allow me also to add that the medical profession is not yet ready to give up the influence and support of this measure which the TEXAS MEDICAL JOURNAL has among its members.

Yours very truly, E. A. WOLDERT,
President Tyler Medical Society.

